# Intelligence artificielle et évaluation des articles scientifiques : promesses et vigilance

**DOI:** 10.48327/mtsi.v6i3.2026.862

**Published:** 2026-08-17

**Authors:** bastien SÉLARRÉCHÉ

**Affiliations:** Service de biologie médicale, Hôpital national d’instruction des armées Bégin, 69 avenue de Paris, 94160 Saint-Mandé, France École du Val-de-Grâce, Académie de santé des armées, 1 place Alphonse Laveran, 75005 Paris, France UMR-S1144, Université Paris Cité, 4 avenue de l’Observatoire, 75006, Paris, France

**Figure:**
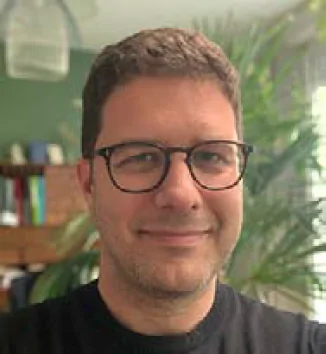

Dans son éditorial d’avril 2026, Jean-Philippe Chippaux nous alertait sur les limites de l’intelligence artificielle (IA) générative utilisée à des fins de rédaction d’articles scientifiques [1]. Cependant, les auteurs ne sont pas les seuls concernés par cette révolution qui s’impose progressivement dans l’ensemble de l’écosystème de la publication scientifique. Les revues médicales sont désormais confrontées à une question centrale : comment intégrer ces outils sans compromettre la qualité, l’indépendance et l’intégrité du processus éditorial ?

Les opportunités offertes par l’IA dans l’évaluation de manuscrits sont multiples, mais concernent des fonctions très différentes. Dans un contexte de difficulté croissante à recruter des relecteurs, elle peut contribuer à identifier des experts pertinents et à détecter d’éventuels conflits d’intérêt. D’autres outils assistent les éditeurs dans la vérification des références bibliographiques ou de la conformité d’un manuscrit aux recommandations internationales (CONSORT, STROBE, STARD, PRISMA, …). Certains modèles sont capables d’identifier des incohérences méthodologiques ou statistiques tandis que d’autres contribuent à la détection du plagiat, d’images manipulées ou de contenus générés par IA. L’IA peut également apporter une aide linguistique en améliorant la qualité rédactionnelle des rapports d’évaluation ou en reformulant des commentaires éditoriaux. Enfin, elle peut faciliter le travail éditorial en produisant une synthèse des avis des relecteurs afin d’éclairer la décision finale d’acceptation ou de refus d’un manuscrit [2].

Des expériences récentes menées dans des revues internationales ont montré qu’un processus hybride associant relecteurs humains et assistance par IA pouvait raccourcir significativement les délais d’évaluation. Ainsi, NEJM AI est à présent capable de rendre une première décision éditoriale en moins de sept jours [4]. L’IA générative est particulièrement performante pour les tâches répétitives ou techniques : vérification de tableaux, cohérence entre protocole et analyse statistique, repérage d’omissions méthodologiques ou reformulation de commentaires éditoriaux.

Dans un environnement où les délais de publication deviennent un enjeu stratégique, ces gains d’efficacité ne peuvent être ignorés.

Cependant, ces différentes fonctions ne présentent, ni le même niveau de maturité, ni le même degré de fiabilité. Les performances observées dépendent largement des plateformes utilisées, des données sur lesquelles elles ont été entraînées et des modalités de leur déploiement. Les tâches relativement standardisées, comme certaines vérifications formelles ou linguistiques, sont généralement plus robustes que celles nécessitant une interprétation scientifique complexe, une appréciation méthodologique fine ou un jugement éditorial.

Les modèles génératifs ne raisonnent pas comme des experts scientifiques; ils produisent des réponses probabilistes fondées sur des *corpus* d’entraînement parfois incomplets, biaisés ou obsolètes. L’intégration d’articles scientifiques de faible qualité dans les *corpus* d’apprentissage peut progressivement dégrader les performances des modèles, créant un véritable cercle vicieux. Lorsque l’IA est utilisée dans le cadre du processus éditorial, elle devrait donc reposer sur des environnements sécurisés, privés et maîtrisés par les institutions. La reproductibilité reste insuffisante, le téléversement d’un même manuscrit à plusieurs reprises peut conduire à des rapports d’évaluation différents, voire contradictoires.

La question de la confidentialité est également cruciale. Le processus d’évaluation par les pairs repose sur la protection des manuscrits non publiés et sur la confidentialité des échanges entre auteurs, relecteurs et éditeurs. Téléverser un manuscrit dans un outil d’IA externe peut exposer des données scientifiques inédites, voire des informations sensibles. Cette problématique est particulièrement importante dans le champ de la santé internationale, où les travaux concernent souvent des populations vulnérables et des données issues de terrains sensibles.

Au-delà des enjeux techniques, l’IA soulève une interrogation plus fondamentale sur la nature même de l’évaluation scientifique. La relecture d’un article ne consiste pas uniquement à identifier des erreurs ou à vérifier des critères méthodologiques. Elle mobilise également une expertise contextuelle, une appréciation de la pertinence clinique ou de santé publique, ainsi qu’une capacité à évaluer l’originalité et l’impact potentiel des travaux. Ces dimensions reposent encore largement sur le jugement humain.

Le risque de biais d’automatisation, du fait d’une confiance excessive dans les recommandations d’origine algorithmique, doit également être pris en compte. La pression éditoriale et la multiplication des soumissions pourraient conduire à la tentation de déléguer progressivement certaines décisions à des systèmes automatisés. Or les performances des outils d’IA sont très hétérogènes selon les fonctions considérées : si certaines applications apportent une aide utile, d’autres restent insuffisamment fiables ou soulèvent des questions éthiques importantes. Une telle évolution pourrait conduire à une standardisation de l’évaluation scientifique et à une réduction de la diversité des approches méthodologiques ou intellectuelles. Il y a là un réel risque d’érosion de l’expertise humaine.

Pour ces raisons, plusieurs grandes maisons d’édition scientifique ont adopté des politiques restrictives concernant l’usage de l’IA dans l’évaluation par les pairs. Certaines interdisent explicitement le téléversement de manuscrits dans des outils génératifs; d’autres autorisent un usage limité et transparent, à condition que la responsabilité finale demeure entièrement humaine [3]. Un consensus semble aujourd’hui émerger : l’IA peut être un outil d’assistance, mais ne peut constituer un relecteur autonome.

Dans les revues de santé internationale, ces débats prennent une dimension particulière. Les inégalités d’accès aux ressources scientifiques, les barrières linguistiques et la faible disponibilité de relecteurs spécialisés pourraient rendre les outils d’IA particulièrement attractifs. Ils pourraient contribuer à renforcer l’inclusivité de la publication scientifique et à soutenir des équipes disposant de moyens limités. Mais ces bénéfices potentiels ne doivent pas masquer les risques d’une dépendance technologique accrue, ni les biais potentiellement intégrés dans des modèles majoritairement développés dans des contextes occidentaux [5].

L’enjeu des prochaines années ne sera probablement pas de choisir entre intelligence humaine et intelligence artificielle, mais d’organiser une collaboration raisonnée entre les deux. Les outils génératifs peuvent améliorer certaines dimensions du travail éditorial, à condition d’être utilisés avec transparence, esprit critique et supervision humaine constante. Les revues scientifiques ont tout intérêt à privilégier des solutions institutionnelles sécurisées, fonctionnant dans des environnements garantissant la confidentialité des manuscrits et la protection des données. Il est donc nécessaire dès à présent pour *Médecine Tropicale et Santé Internationale* de définir des règles claires pour protéger l’intégrité de l’évaluation par les pairs et promouvoir un usage responsable de l’IA.
