# Supplementary material for: Artificial intelligence and the evaluation of scientific articles: promise and caution
Source: Med Trop Sante Int. 2026 Aug 17;6(3):mtsi.v6i3.2026.862. [Article in French] doi: 10.48327/mtsi.v6i3.2026.862 (PMC13697902; doi:10.48327/mtsi.v6i3.2026.862)
Supplement: Related texts

# Research article: Artificial intelligence and the evaluation of scientific articles: promise and caution

In his April 2026 editorial, Jean-Philippe Chippaux warned us about the limitations of generative artificial intelligence (AI) used for writing scientific articles [1]. However, authors are not the only ones affected by this revolution, which is gradually taking hold throughout the entire scientific publishing ecosystem. Medical journals are now faced with a central question: how can they integrate these tools without compromising the quality, independence, and integrity of the editorial process?

AI offers numerous opportunities for manuscript evaluation, but these opportunities involve very different functions. Given the growing difficulty in recruiting reviewers, AI can help identify relevant experts and detect potential conflicts of interest. Other tools assist publishers in verifying bibliographic references or ensuring a manuscript’s compliance with international guidelines (CONSORT, STROBE, STARD, PRISMA, etc.). Some models are capable of identifying methodological or statistical inconsistencies, while others help detect plagiarism, manipulated images, or AI-generated content. AI can also provide linguistic support by improving the writing quality of review reports or by rephrasing editorial comments. Finally, it can streamline the editorial process by generating a summary of reviewers’ opinions to inform the final decision to accept or reject a manuscript [2].

Recent experiments published in international journals have demonstrated that a hybrid process combining human reviewers and AI assistance can significantly reduce review times. For instance, NEJM AI can now render an initial editorial decision in under seven days [4]. Generative AI is particularly effective at repetitive or technical tasks, such as verifying tables, ensuring consistency between the protocol and statistical analysis, identifying methodological omissions, and rephrasing editorial comments. In an environment where publication timelines are becoming a strategic issue, these efficiency gains cannot be ignored.

However, these functions vary in maturity and reliability. Observed performance largely depends on the platforms used, the data on which they were trained, and the terms of their deployment. Tasks that are relatively standardized, such as certain formal or linguistic checks, are generally more robust than tasks requiring complex scientific interpretation, nuanced methodological assessment, or editorial judgment.

Generative models do not reason like scientific experts; they produce probabilistic responses based on training corpora that are sometimes incomplete, biased, or obsolete. Including low-quality scientific articles in training corpora can gradually degrade model performance, creating a vicious cycle. Therefore, when AI is used as part of the editorial process, it should be based on secure, private environments controlled by institutions. Reproducibility remains insufficient: uploading the same manuscript multiple times can result in different—or even contradictory—evaluation reports.

The issue of confidentiality is also crucial. The peer-review process depends on protecting unpublished manuscripts and maintaining the confidentiality of communications between authors, reviewers, and editors. Uploading a manuscript to an external AI tool can expose unpublished scientific data or sensitive information. This issue is particularly significant in global health research, which often involves vulnerable populations and data from sensitive settings.

Beyond technical challenges, AI raises a more fundamental question about the nature of scientific evaluation itself. Peer review of an article is not merely about identifying errors or verifying methodological criteria. It also requires contextual expertise, an assessment of clinical or public health relevance, and the ability to evaluate the research’s originality and potential impact. These aspects still rely heavily on human judgment.

The risk of automation bias, due to excessive reliance on algorithm-generated recommendations, must also be considered. Editorial pressure and the increasing number of submissions could lead to the temptation to gradually delegate certain decisions to automated systems. However, AI tool performance varies greatly depending on the specific function: some applications provide useful assistance, while others are insufficiently reliable or raise significant ethical questions. This could lead to the standardization of scientific evaluation and reduce the diversity of methodological and intellectual approaches. There is a real risk of eroding human expertise here.

For these reasons, several major scientific publishers have adopted restrictive policies regarding the use of AI in peer review. Some explicitly prohibit uploading manuscripts to generative tools, while others allow limited, transparent use provided that humans remain entirely responsible [3]. A consensus is now emerging: AI can serve as a support tool but cannot function as an autonomous reviewer.

In international health journals, these debates take on a particular dimension. Inequalities in access to scientific resources, language barriers, and the limited availability of specialized reviewers could make AI tools appealing. These tools could enhance the inclusivity of scientific publishing and support teams with limited resources. However, the potential benefits must not obscure the risks of increased technological dependence or the biases that may be embedded in models developed primarily in Western contexts [5].

In the coming years, the challenge will likely not be choosing between human and artificial intelligence, but rather organizing a thoughtful collaboration between the two. Provided they are used with transparency, critical thinking, and constant human oversight, generative tools can improve certain aspects of editorial work. Scientific journals should prioritize secure institutional solutions operating in environments that guarantee manuscript confidentiality and data protection. Therefore, it is necessary for *Médecine Tropicale et Santé Internationale* to establish clear rules to protect the integrity of peer review and promote the responsible use of AI now.
